# Spatial Analysis of COVID-19 Vaccine Centers Distribution: A Case Study of the City of Jeddah, Saudi Arabia

**DOI:** 10.3390/ijerph19063526

**Published:** 2022-03-16

**Authors:** Kamil Faisal, Sultanah Alshammari, Reem Alotaibi, Areej Alhothali, Omaimah Bamasag, Nusaybah Alghanmi, Manal Bin Yamin

**Affiliations:** 1 Geomatics Department, Faculty of Architecture and Planning, King Abdulaziz University, Jeddah 21589, Saudi Arabia; 2 Department of Computer Science, Faculty of Computing and Information Technology, King Abdulaziz University, Jeddah 21589, Saudi Arabia; aalhothali@kau.edu.sa (A.A.); obamasek@kau.edu.sa (O.B.); 3 Department of Information Technology, Faculty of Computing and Information Technology, King Abdulaziz University, Jeddah 21589, Saudi Arabia; ralotibi@kau.edu.sa (R.A.); nayyadahalghanmi@stu.kau.edu.sa (N.A.); 4 Planning and Transformation Department, Ministry of Health, Jeddah 21176, Saudi Arabia; mabinyamin@moh.gov.sa

**Keywords:** COVID-19, vaccine, vaccination centers, GIS analysis, spatial analysis, Saudi Arabia, Jeddah

## Abstract

The COVID-19 pandemic is one of the most devastating public health emergencies in history. In late 2020 and after almost a year from the initial outbreak of the novel coronavirus (SARS-CoV-2), several vaccines were approved and administered in most countries. Saudi Arabia has established COVID-19 vaccination centers in all regions. Various facilities were selected to set up these vaccination centers, including conference and exhibition centers, old airport terminals, pre-existing medical facilities, and primary healthcare centers. Deciding the number and locations of these facilities is a fundamental objective for successful epidemic responses to ensure the delivery of vaccines and other health services to the entire population. This study analyzed the spatial distribution of COVID-19 vaccination centers in Jeddah, a major city in Saudi Arabia, by using GIS tools and methods to provide insight on the effectiveness of the selection and distribution of the COVID-19 vaccination centers in terms of accessibility and coverage. Based on a spatial analysis of vaccine centers’ coverage in 2020 and 2021 in Jeddah presented in this study, coverage deficiency would have been addressed earlier if the applied GIS analysis methods had been used by authorities while gradually increasing the number of vaccination centers. This study recommends that the Ministry of Health in Saudi Arabia evaluated the assigned vaccination centers to include the less-populated regions and to ensure equity and fairness in vaccine distribution. Adding more vaccine centers or reallocating some existing centers in the denser districts to increase the coverage in the uncovered sparse regions in Jeddah is also recommended. The methods applied in this study could be part of a strategic vaccination administration program for future public health emergencies and other vaccination campaigns.

## 1. Introduction

Due to the rapid spread of the novel coronavirus disease (COVID-19), caused by the novel severe acute respiratory syndrome coronavirus 2 (SARS CoV-2), governments worldwide started to take action to contain this pandemic. Within a year from the start of the pandemic in March 2020 [1], the cumulative numbers of the reported COVID-19 cases reached over 79 million in December 2020, with over 1.7 million deaths globally [2]. Severe preventive measures were implemented to slow the spread of the disease across the world, including travel restrictions, school closures, public lockdowns, and curfews. With the uncertainty regarding the virus’ epidemiology and the absence of valid vaccines, countries were elevating and alternating between these various strict non-pharmaceutical measures.

Within the first few months of the spread of the novel coronavirus disease, several test types and testing protocols for COVID-19 were approved and implemented [3]. Hence, widespread testing for COVID-19 was proven to be an effective measure applied by most countries. To maximize testing capacity, the testing for COVID-19 was administered via medical facilities and drive-through and walk-in testing stations [4]. Mass testing, as considered by the World Health Organization (WHO), is the best method to slow the spread of the virus [5]. However, the development of a vaccine has been an essential step in controlling this highly contagious disease.

Fortunately, after almost two years from the initial COVID-19 outbreak in December 2019 and with international vaccine-development efforts, the approval for the emergency use of the first mRNA COVID-19 vaccine, Pfizer–BioNTech, was issued by the U.S. Food and Drug Administration (FDA) on 11 December 2020 [6]. Since then, numerous vaccines have been developed and approved, including Oxford–AstraZeneca, Moderna, and Johnson & Johnson [7]. To combat the pandemic and protect high-risk groups, most countries began initiating vaccination campaigns based on various vaccination strategies and used one or more licensed COVID-19 vaccines.

**Figure 1:**
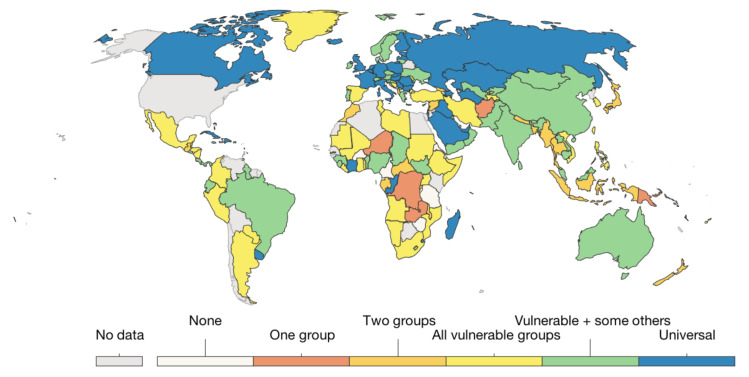
COVID-19-vaccine-delivery policies based on the number of the targeted vulnerable groups per country [9].

According to the vaccine supply and the size of the population, public health authorities in most countries prioritize the distribution of the vaccine by targeting high-risk groups, such as healthcare workers, the elderly (over the age of 65 years), and individuals with medical conditions who are more likely to die or suffer severely from COVID-19. Additionally, as recommended by WHO [8], high-risk populations should be prioritized for COVID-19 vaccinations to minimize the complications and deaths caused by COVID-19. Figure 1 illustrates the distribution of the different vaccination policies worldwide [9], where countries target one or more vulnerable groups.

During health emergencies and disease outbreaks, such as the COVID-19 pandemic, it is important to ensure a timely and equitable administration of medical care and health services for all individuals in the affected community. A successful emergency response requires identifying and managing locations or facilities accessible to the entire population to deliver basic needs and health services at these points. Spatial analysis and geographic information systems (GIS) technology can be successfully applied to assess the equitable allocation of sites for PODs (points of dispensing), where medications, vaccinations, or other health services can be provided for a large number of people during public health emergencies.

Since the discovery of COVID-19 and its rapid spread across the world, spatial analysis has played a significant role in the study and mapping of the spread of the disease [10,11]. Several GIS tools and spatial-analysis techniques have been used to track COVID-19 at multiple levels [12,13,14,15,16]. Researchers have also studied the spatial distribution of COVID-19’s incidence and mortality rates in relation to various aspects such as age, gender, and living conditions [17,18]. As widespread testing has become one of the main preventative measures in many countries to reduce the transmission of COVID-19, studies of the spatial distribution of the epidemic’s progression and its correlation with the testing rates were conducted [19].

In the case of mass COVID-19 vaccinations and the scale of the expected coverage, GIS tools can help in planning the allocation of vaccine sites based on a set of potential candidate locations that can serve as vaccination hubs. GIS technology can map the vulnerable populations who might have problems reaching vaccination centers, such as people in rural or remote areas [20,21]. Additionally, GIS analysis can be used to identify vaccination rates in different geographical areas to ensure the fair and efficient distribution of the vaccination centers [22]. Several research studies have focused on the spatial analysis of COVID-19-vaccine administration in various countries and regions to assess the vaccination rates and coverage [23]. GIS technology was also applied to optimize the distribution of COVID-19 vaccines to vaccination centers [24].

The first confirmed case of COVID-19 in Saudi Arabia was reported on 2 March 2020 [25]. In Jeddah, a major city in Saudi Arabia, the initial cases of COVID-19 were detected on 11 March 2020. According to the Department of Infectious Diseases at the Directorate of Health Affairs in Jeddah, during the rapid spread of the COVID-19 outbreak in Saudi Arabia, the highest number of confirmed COVID-19 cases reported in Jeddah was 20,069 by the end of August 2020. Along with other preventative measures, such as limiting public gatherings, lockdowns, and curfews, enforced throughout the country, large-scale COVID-19 testing effectively contributed to the containment of the outbreak. Several fixed and mobile COVID-19-testing centers were established in Jeddah by the end of July 2020.

Saudi Arabia was one of the first countries to establish COVID-19 vaccination administration on 17 December 2020 using Pfizer–BioNTech, AstraZeneca, and, more recently, Moderna, with a target to vaccinate 70% of the adult population, including citizens and residents in the country [26]. Close to the end of 2021, more than 65% of the population of Saudi Arabia was fully vaccinated, with 48 million doses administered. Due to being one of the major cities with a high population, Jeddah was prioritized in the first phase of the vaccine-distribution plan in Saudi Arabia. The vaccination centers in Jeddah include public and private healthcare facilities, old airports, stadiums, and shopping malls.

The goal of this study was to analyze the spatial distribution of the COVID-19 vaccination centers in Jeddah. In particular, it aimed to examine vaccination centers in Jeddah using GIS tools and methods to provide insight into the effectiveness of the current locations of the COVID-19 vaccination centers in terms of accessibility and coverage. An evaluation of the spatial distribution of the COVID-19 vaccination sites will facilitate the decision-making process for adjusting the number and locations of these designated centers to fulfill the expected vaccination rates for the entire population in Jeddah.

The rest of the paper is organized as follows: Section 2 provides details on the area under study, the collected data, and the GIS methods applied to achieve the objective of our study. In Section 3, the results obtained are presented and analyzed. Finally, we conclude, in Section 4, with a discussion and future directions.

## 2. Materials and Methods

### 2.1. Study Area

**Figure 2:**
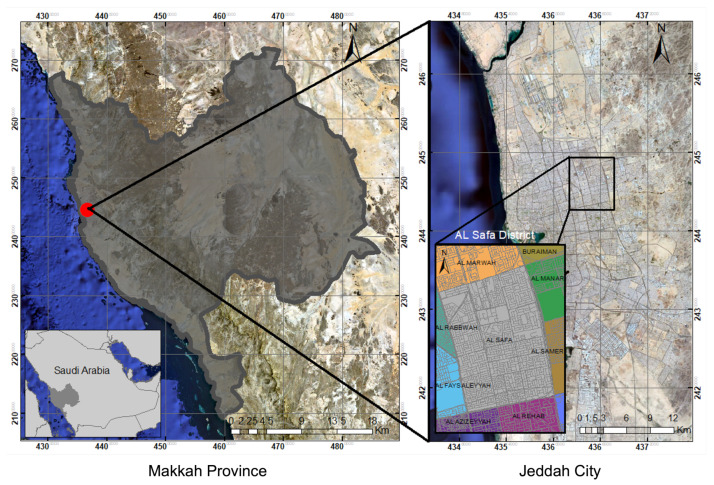
Location map of the city of Jeddah.

Jeddah is one of the major cities in the region of Makkah, which is one of the 13 administrative provinces of Saudi Arabia. The city of Jeddah is located in the western region of the Kingdom (Figure 2); its central location is longitude E $39^{\circ }11^{\prime }52.69^{\prime \prime }$ and latitude N $21^{\circ }32^{\prime }32.57^{\prime \prime }$, at an elevation of 15 m above sea level [27]. The geographical area of the province is approximately 1765 km${}^{2}$. In 2016, the population of Jeddah was around 4.08 million, accounting for one-eighth of the entire population of Saudi Arabia. It is now the country’s second-largest metropolis after the capital city Riyadh. Over time, the city’s services have grown in several sectors such as transportation, communication, health initiatives, and public utilities, including water, power, and other utilities [28].

The annual population growth rate has risen to 3.5%, mainly due to job opportunities in the city as well as its proximity to the busiest international airport, the largest seaport in the region, and the Holy City of Makkah [28]. The average household size in Jeddah is 5.2 people, and 41% of the population is under the age of 23, with only 3% being 65 or older. Health services are supplied by multiple sectors, including the Ministry of Health’s network of primary healthcare facilities and hospitals across the city, in addition to other public institutions and the private sector [29].

### 2.2. Data

In addition to being one of the major cities in Saudi Arabia, Jeddah was chosen for this study mainly due to the availability of the required data and the significant population expansion over the last decade. The data acquired from the Jeddah Municipality and used in this study include population and district data. The numbers and locations of the COVID-19 vaccination centers in Jeddah were obtained from the published data provided by the Saudi Ministry of Health (MoH) in 2020. These data for the vaccination centers were updated for the additional vaccination centers established in 2021. All collected data were first imported into the ArcGIS platform and then projected to the Universal Transverse Mercator (UTM) 37 N coordinate system for further investigation. The data were preprocessed using a GIS environment to remove outliers, yielding a new weighted point feature class, used as input for the subsequent spatial-analysis phase. Table 1 provides a summary of the data used in this research work.

### 2.3. Analytical Approach

**Figure 3:**
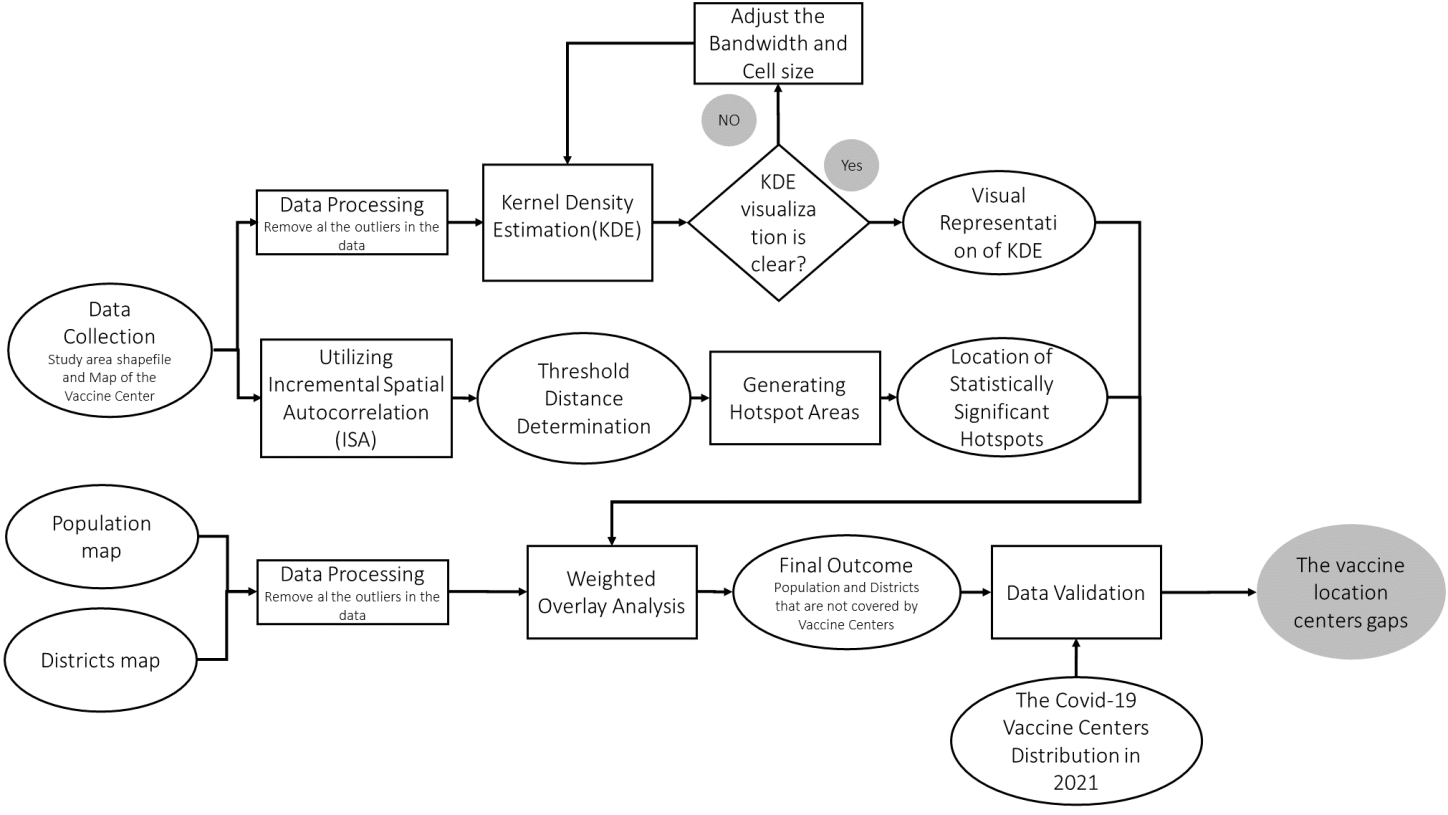
The workflow of the applied methodology.

The methodology of this research work consists of five steps. First, spatial data were first imported into ArcGIS (ArcGIS; Esri; Redlands, CA, USA) for data processing. Then, in the second step, kernel density estimation (KDE) was employed to determine the hotspot locations of the vaccination centers established in 2020 in Jeddah. KDE is a powerful GIS approach used to comprehend and potentially indicate the patterns of a spatial event [30]. In the third step, incremental spatial autocorrelation (ISA) was used to measure the strength of spatial clustering for each distance of the vaccination centers. Next, we used hotspot analysis (Getis–Ord) to determine the statistical significance of the hotspots and assess their incident intensity according to the problem under study. Then, in the final step, the population and district maps were used to determine the total number of districts that were not served by the vaccination centers. Further analysis was performed for additional vaccination centers established in 2021 to compare the spatial distribution of the COVID-19 vaccination centers in 2020. Figure 3 illustrates the workflow for the proposed methodology in this research work. The following sections provide a detailed description for each step of the methodology.

#### 2.3.1. Kernel Density Estimation (KDE)

Kernel density estimation is a statistical method that is classified as a technically advanced approach in point–pattern analysis. KDE is generally used to map and evaluate hotspots, which helps in the visualization of results. The main purpose of KDE is to comprehend and potentially indicate the patterns of a spatial event [30]. By implementing the KDE technique, the entire region was first split into a set number of cells [31]. Spatial analysis using KDE represented the intensity of specific areas to identify the study area’s spatial pattern densities, including high- and low-density regions [32]. The density was computed by superimposing a smooth Gaussian curve over each zone and by calculating the distances between the observation and the reference points (locations of interest) using a mathematical formula known as the kernel function. The reference points in this study mainly represent the locations of COVID-19 vaccination centers.

The KDE technique calculates the density for each zone or district by changing the values within the search radius for that reference point. The density is the greatest at the reference point location, reduces with distance, and converges to zero at the specific radius when the distance decay effect is taken into consideration [31]. The following Formula (1) is the general equation for KDE [31]:

(1) $$\lambda_{\tau }\left(s\right)=\sum_{i=1}^{n}\frac{1}{\tau^{2}}k\left(\frac{s-s_{i}}{\tau }\right)$$

where $\lambda_{\tau }\left(s\right)$ is density evaluation at position *s*, *n* is the $\lambda_{\tau }\left(s\right)$ number of positions or locations, $s_{i}$ is the (x,y) position of the $i_{th}$ vaccination center, τ is the bandwidth, and *k* is the kernel algorithm, which is a function of the bandwidth and distance [33]. Various spatial-analysis studies have proposed different kernel functions, such as Gaussian functions [34,35,36].

Regardless of the nature of the kernel function, the selection of the suitable grid size and bandwidth is significant for calculating accurate results [35,37]. An undersmoothed density map can be generated using a narrow bandwidth, which is useful for detecting local impacts, but a high bandwidth produces a smoother density estimate with less fluctuation. Hence, an appropriate cell size and bandwidth must be determined for the study depending on the computational time requirements, sample size, and data [38,39,40,41,42].

The bandwidth and cell size are the two most important elements that influence KDE analysis. The KDE’s output is often shown in a raster dataset, which consists of a grid of pixels. To simulate a cluster that is observed in reality, the cell size must be appropriately determined. In addition, the size of the cluster depends on the computational time, sample size, and amount of data to store. Larger grid cell sizes speed up processing; however, information is more likely to be averaged over a larger area, which may lead to oversmoothing and the loss of valuable hotspot information. On the other hand, selecting a small grid size may make it time consuming, rendering the analysis process burdensome.

#### 2.3.2. Incremental Spatial Autocorrelation (ISA)

The spatial connection model used in this study to estimate $G_{i}^{*}$ is the fixed distance band (FDB), which uses an appropriate distance (threshold) to select which areas are to be included. To establish an appropriate threshold distance, we used the ISA method. The Spatial Autocorrelation (Global Moran’s I) tool was used to employ the ISA technique in the GIS to assess the intensity of spatial clustering throughout a sequence of increasing distances. Moran’s I index is a spatial autocorrelation coefficient. A Moran’s I around +1 denotes clustering, whereas a number near −1 denotes dispersion. Z-scores and p-values were calculated to assess the statistical significance of the Moran’s I value [43]. The Z-score indicated clustering intensity. The ISA process is as follows:

1. To estimate the spatial autocorrelation, first, a default minimum distance is chosen. The ISA technique then evaluates the distance between features to ensure that each segment has a neighbor. In this research, 2 km (kilometers) was set as the default distance for the ISA technique. The spatial autocorrelation values are then computed by increasing the distance incrementally.
2. The ideal threshold distance is the one with the highest Z-score. The threshold distance in this investigation was determined to be 2 km. This determined distance was utilized in the spatial mechanisms to generate clustering in the data.

Moran’s I index is denoted as follows [31]:

(2) $$I=\frac{\sum_{i=1}^{n}\sum_{j=1}^{n}W_{ij}\left(X_{i}-\bar{X}\right)\left(X_{j}-\bar{X}\right)}{\sigma^{2}\sum_{i=1}^{n}\sum_{j=1}^{n}W_{ij}}$$

where $X_{i}$ and $X_{j}$ refer to the number of variables at vaccination locations *i* and adjacent locations *j*. *n* refers to the numbers of vaccine locations, and $W_{ij}$ is a set row of standardized weights, representing associations between locations *i* and nearby locations *j*. σ represents variance, which can be calculated as follows.

(3) $$\sigma^{2}=\frac{\sum_{i=1}^{n}{\left(X_{i}-\bar{X}\right)}^{2}}{n}$$

The threshold distance acquired was then used in the hotspot technique (Getis–Ord) to determine the statistical significance of the hotspots detected by KDE.

#### 2.3.3. Hotspot Analysis

Getis and Ord (1992) developed the $G_{i}^{*}$ statistical approach for detecting statistically significant geographical groupings of high value, i.e., hot spots, and low values as coldspots. The $G_{i}^{*}$ statistic indicates the degree of association between a concentration of regression points and the rest of the event points based on a specific radius or threshold. It is not uncommon for KDE and $G_{i}^{*}$ to provide visually identical results. The kernel density function and the Getis–Ord $G_{i}^{*}$ statistic, on the other hand, are two entirely separate analyses. While KDE tries to find high-value clusters in data, the $G_{i}^{*}$ statistic not only finds but also understands the geographic clusters of the phenomena under investigation [32]. The following equation represents the $G_{i}^{*}$ statistic [31].

(4) $$G_{i}^{*}\left(d\right)=\frac{\sum_{j}w_{ij\left(d\right)x_{j}}}{\sum_{j}x_{j}}\;\;\;\;where\;j\neq i$$

$G_{i}^{*}$ is a number that describes the spatial dependence of the location *i* across all n vaccination positions, *d* is the assigned distance, $x_{j}$ is the number of vaccination centers at position *j*, and $w_{ij}$ is a spatial weight that represents the association between two vaccination center positions, *i* and *j*, using the fixed distance band [31].

The hotspot method was utilized to perform the $G_{i}^{*}$ statistical significance test. This technique determines whether a cluster of high-intensity data is surrounded by high-intensity values. A coldspot occurs when low-intensity values surround low-intensity values. The statistical significance test provides $G_{i}^{*}$z scores and $G_{i}^{*}$p values. A high positive $G_{i}^{*}$z score represents high-value clusters, indicating hotspot areas. A lower negative *z* score indicates a concentration of low values, known as coldspots. A $G_{i}^{*}$z near 0 denotes a random distribution [31].

#### 2.3.4. Hotspot Selection Criteria

**Figure 4:**
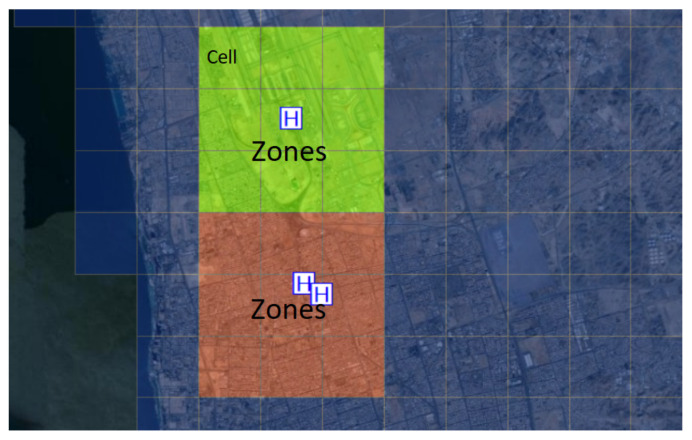
An illustration of the spatial distribution of the cells and zones in a geographical area, where the letter H indicates medical facilities or vaccination centers.

The outcomes take the form of a color-coded map. There is no rule of thumb or set of specific values to use as a guideline in evaluating whether regions are hotspots or not. A subjectively determined cut-off value can be used to screen for places with comparatively high risk throughout a study region. As an example from a different context, let us consider the overall average of the estimated production output as the cut-off. When the predicted production output at a given place exceeds this threshold, the site is then designated as a hotspot. In the real world, however, other factors (the availability of financial resources, policies, and regulations) might be at play, affecting the production activities at different groups of sites. An alternate approach utilized in this study is the quantile approach, which classifies the predicted values into distinct groups. In this approach, groups of zones are generated and divided evenly across the classifications, resulting in an equal number of grid cells in each zone, as shown in Figure 4. Each cell indicates the number of locations (e.g., locations of medical facilities or vaccination centers) [44,45]. The hotspots are subsequently identified as the spots with the highest numbers of locations of interest—for this study, the COVID-19 vaccination centers.

#### 2.3.5. Weighted Overlay

**Figure 5:**
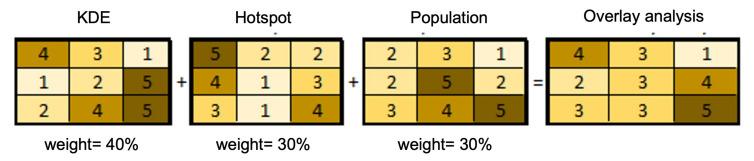
An example of the overlay analysis of selected layers.

The weighted overlay uses numerous layers to solve the multicriteria issues using a standard measurement scale according to importance. After preparing input layers, including the population distribution map, KDE map, and hotspot distribution map, all layers were then reclassified in the same scale. Figure 5 provides an example of the weighted overlay of selected layers in this study. The weighted overlay was performed for all layers. Given the weighted overlay, we then generated the output map, i.e., the spatial distribution of the COVID-19 vaccination centers across the city of Jeddah.

## 3. Results and Discussion

### 3.1. Spatial Analysis of COVID-19 Vaccination Sites in the Year 2020 in Jeddah

**Figure 6:**
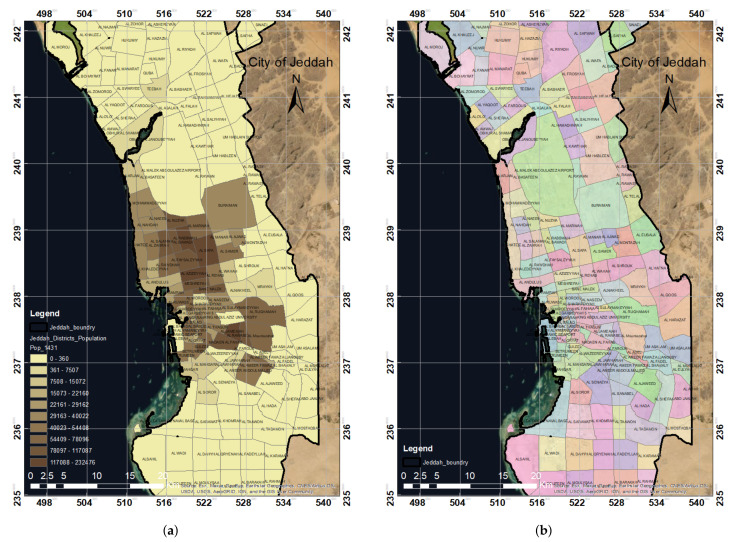
The spatial distribution of the population in Jeddah by district (**a**) and a map of districts in the city of Jeddah (**b**). (**a**) Population distribution map. (**b**) District distribution map.

The population map in Figure 6a represents the population distribution in Jeddah city based on the data collected in this study. The colored or shaded areas in the map represent the distribution of the population at the district level, where the green area has the highest population (up to 230,000) and the white-colored area has the lowest population (up to 23,000). Based on the spatial analysis of the locations of the vaccination centers in Jeddah, the population map was used to determine the number of people not covered by the vaccination centers. The district map in Figure 6b illustrates the officially listed neighborhoods of Jeddah city. Using the district map, we were able to represent the coverage of the vaccination centers based on the location of these centers at the district level. This will help in identifying which districts are fully covered by the vaccination centers and which districts need more vaccination centers.

**Figure 7:**
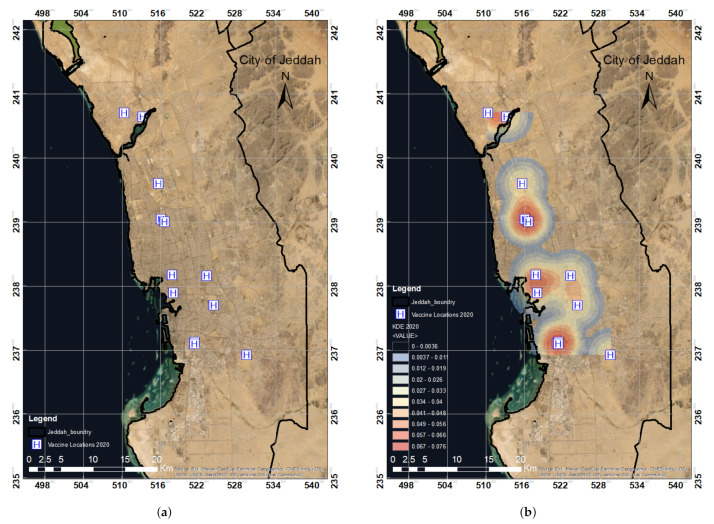
The spatial distribution of COVID-19 vaccination centers in 2020 in Jeddah (**a**) and the KDE analysis over all the initial 12 vaccination locations in Jeddah in 2020 (**b**). (**a**) Spatial distribution map of the vaccination centers in 2020. (**b**) KDE analysis of vaccine locations in 2020.

The locations of the vaccination centers in 2020 were spatially digitized using the ArcGIS platform, as shown in Figure 7a. Density was high at the vaccination centers’ locations, as shown in Figure 7b. The KDE value decreased with distance and converged to zero at a radius of 2000 m. The spatial locations of the vaccination centers were then analyzed using KDE and hotspot distribution analysis by considering population levels and districts.

KDE analysis (Figure 7b) was conducted for the vaccination centers listed in 2020 in the city of Jeddah. To determine the density of the point features and their surrounding raster data, we utilized the kernel density for the point features tool. The tool’s algorithm fitted a nicely curved surface over each point (vaccination center location). As described in Section 2.3.1, the bandwidth and size influence the KDE results as these two parameters determine the size of the search region. The bandwidth of the normal kernel function is equal to the normal distribution’s standard deviation. The radius of the interpolated search area is the bandwidth of the triangular, quartic, uniform kernels. The choice of bandwidth is entirely subjective [39,46,47,48]. A smaller bandwidth interval frequently results in an acceptable mesh density covering all higher and lower levels; a wider bandwidth interval produces a flatter distribution and, consequently, is more likely to allow the detection of minor variations between locations. Although smaller bandwidths indicate more distinction across regions, the statistical accuracy of the estimate must also be taken into account. Brimicombe [49] suggested a bandwidth that is six, nine, or twelve times the median distance between nearest neighbors.

Overall, experimenting with fixed intervals to determine which findings make the most sense is recommended [46]. Previously, numbers ranging from 20 to 1000 m (meters) were utilized in different studies [35,38,40,50,51]. In our study, we considered two different bandwidth levels: 2000 and 2500 m. The bandwidth sensitivity of the hotspot pattern was examined at 2000 m. As mentioned previously, the density value of the surface is highest at the regression point and drops as one moves away from it. At the search radius distance (bandwidth) from the location, density converged to zero. In the absence of user input, the program automatically determines bandwidth based on the provided dataset. The search radius units are dependent on the linear unit of the output spatial reference’s projection.

**Figure 8:**
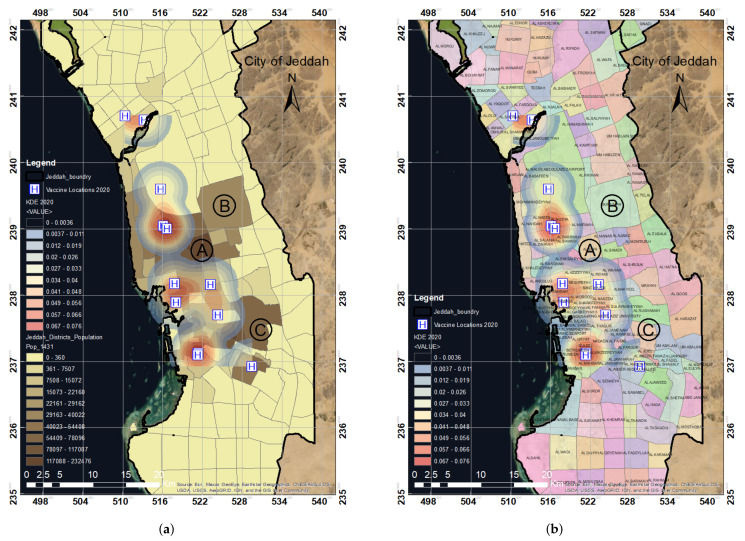
The overlay analysis of KDE with respect to (**a**) population data and (**b**) district data based on the spatial distribution of the COVID-19 vaccination centers established in 2020 in the city of Jeddah. The A, B, and C zones highlight the highly populated regions. (**a**) KDE analysis map in relation to population data. (**b**) KDE analysis map in relation to district data.

A total of 12 vaccination centers were observed in our study area of the Jeddah districts as shown in Figure 8b. It was observed that one center almost covered a 3–5 km radius around it. Due to the limited number of centers, many people lack access to vaccines, and the vaccination centers did not cover the study area we selected. Some people do not even have any access to vaccination centers due to the high volume of individuals. The blue and light-blue areas are those within a 2 km radius where people can obtain vaccines. The color red denotes medium coverage within a 3 km radius; similarly, the light-yellow and yellow colors denote places that are 5 km or more away from vaccination facilities.

Overlay analysis was performed using the KDE output according to the locations of the vaccination centers in Jeddah in 2020 with the population and district distribution. The results show that most populated regions were not covered by vaccination centers, as shown in Figure 8a,b, zones A, B, and C. Similarly, numerous districts were not covered by the 12 vaccination centers. The zones A, B, and C showed that almost 50% of the study area was not covered based on the distribution of the established vaccination centers.

**Figure 9:**
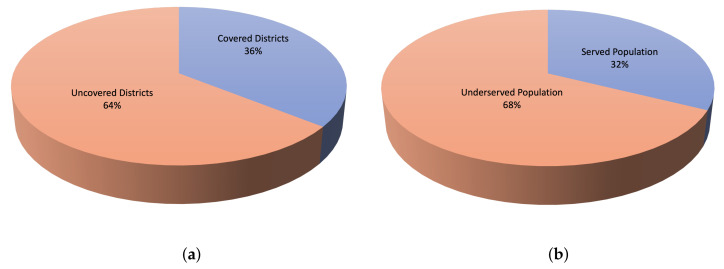
The percentage of (**a**) district coverage and (**b**) population coverage based on the locations of the COVID-19 vaccination centers established in 2020 in the city of Jeddah. (**a**) Percentage of the covered districts. (**b**) Percentage of the served population.

When KDE was overlaid with Jeddah districts, it was observed that, out of 90 districts, only 50 districts were covered or had access to vaccination centers, as shown in Figure 9a. The vaccination centers cover only 36% of the total districts in Jeddah; people from the city’s total population were served. However, more than 2 million people (68% of the total population) were not served, as shown in Figure 9b. The results show that large districts, including Al Safa, Berryman, Al Rughamah, and Al Mountazahat, with populations more than 60,000 people are not covered by the vaccination centers. On the other hand, districts such as South of Obhur, North of Obhur, Al Mohammadeyyah, and Al Naeem with fewer than 40,000 people are among the 36% of the districts covered by the vaccination centers. In summary, it was observed that the set of 2020 vaccination centers was not enough to cover the population of Jeddah. Clearly, there was a need for more vaccination centers, as not all districts and populations were covered, in addition to the shortage of vaccines during the early stages of the vaccine-production ramp-up.

### 3.2. Statistical Significance Analysis for Spatial-Pattern Discovery

Kernel density analysis could show where the clusters in our data were located. However, we could not determine whether these clusters were formed randomly or as a consequence of an underlying spatial mechanism. Therefore, we conducted a subsequent analysis to both discover patterns and assess their significance. This subsequent analysis enabled us to discover underlying patterns, assess their significance, and ensure that conclusions were drawn based on reliable resources other than human visual perception.

The Getis–Ord Gi approach is one of the analytical tools used to determine spatial dispersion. This statistical tool mainly relies on hotspot and coldspot analyses to determine the spatial distribution of data. To check for the hotspots and coldspots in the coverage of the vaccination centers, a hotspot-analysis algorithm was used. We used hotspot analysis to determine the groups of low and high points in the data. With the p- and z-values obtained, we then assessed how statistically significant these clusters were, with 99, 95, or 90 percent confidence.

The main feature (vaccination center location) in the entire dataset was considered in hotspot analysis. This feature is assigned a value when the components are aggregated together. The value represents their count inside the aggregation region. A neighborhood is a collection of elements that surround a feature, including the feature itself. Although a high-value trait is intriguing, it may or may not be statistically significant. A component with a high value must be surrounded by another high-valued feature to be statistically significant. A statistically substantial z-score is obtained when a feature’s sum and neighbors are proportionally associated with the total number of features.

**Figure 10:**
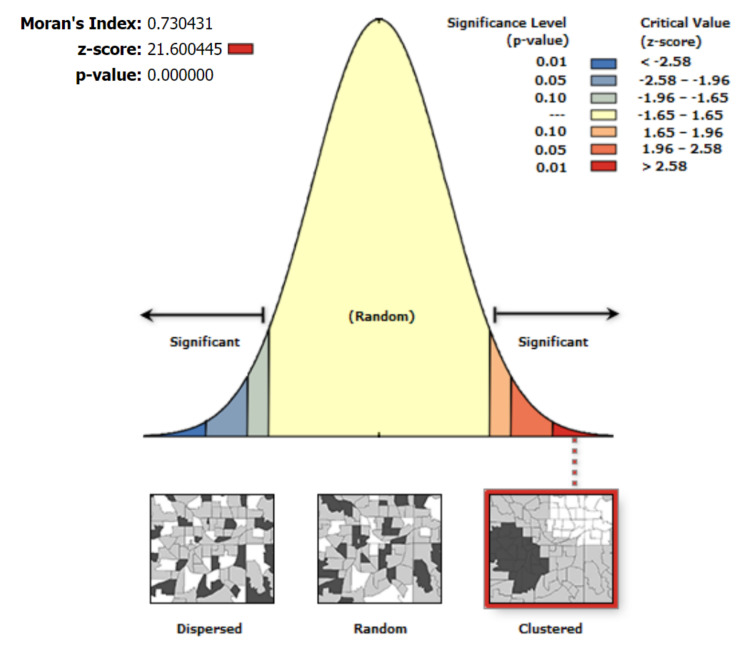
The spatial autocorrelation bell curve for vaccination centers.

A statistically significant z-score is calculated when the local sum differs considerably from the expected local sum. Such a significant difference is considered to be too high to be caused by randomness. When the data have more clustering of high values, the z-score for statistically significant positive z-scores becomes high. The denser the clustering of low values, the smaller the z-score for the statistically significant negative z-scores (coldspot) as shown in Figure 10. Since our dataset was a point-pattern dataset, we utilized hexagonal bins to aggregate the data.

Figure 10 illustrates the spatial autocorrelation bell curve for the vaccination centers. The curve showed clustering and a positive Moran’s I index at 0.7. The results showed a significantly low p-value, which is lower than 0.05. The pattern for the vaccination centers is not random; it has clustered at the assigned threshold distance.

A statistically considerable *z*-score is calculated when the local sum differs significantly from the expected local sum. The difference is too high to be a consequence of random choice. When the data have increased clustering of high values, the *z*-score for statistically significant positive *z*-scores becomes high. The denser the clustering of low values, the smaller the *z*-score for statistically significant negative *z*-scores (coldspot). Due to our data containing points, we utilized hexagonal bins to aggregate them.

**Figure 11:**
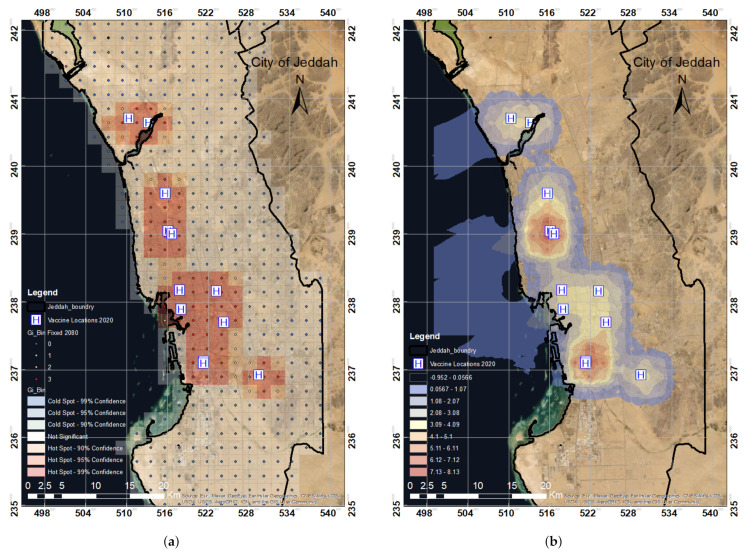
The regions of hotspots and coldspots in the study area (**a**) and the regions of significant and insignificant *z*-score values (**b**) based on the distribution of the COVID-19 vaccination centers established in 2020 in Jeddah. (**a**) Hotspot and coldspot map. (**b**) Spatial *z*-score map.

The main objective of hotspot analysis is to determine if values are high or low. The results of the Getis–Ord Gi approach show that the red area in Figure 11a represents a region of hotspots, implying that this area is covered by vaccination centers. The white region is considered as a coldspot, i.e., an area without vaccination availability. Hotspot density was calculated, as shown in Figure 11b.

Hotspot density analysis is a geographical analysis and mapping approach that focuses on detecting the grouping of spatial phenomena. These spatial phenomena are represented on a map as points that correspond to the locations of events or objects. The regions of nominal values suggest that the lowest densities are dark blue and light blue. The areas with high-density values, i.e., hotspot zones, are red and light red.

**Figure 12:**
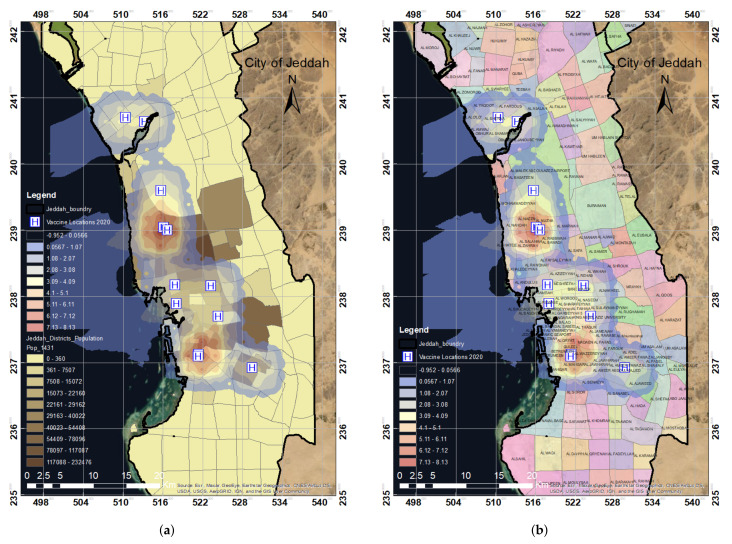
Hotspot density map compared with (**a**) population and (**b**) district data based on the spatial distribution of the COVID-19 vaccination centers established in Jeddah in 2020. (**a**) Hotspot density vs. population. (**b**) Hotspot density vs. districts.

Figure 12a,b shows hotspot density maps compared with district and population data. The results confirmed that several districts, such as Al Safa, Berryman, Al Rughamah, and Al Mountazahat, were not covered by any vaccination center. Based on the location of the vaccination centers, only a few neighborhoods are covered in the city of Jeddah. The hotspot analysis provides almost the exact results of KDE, i.e., showing that vaccination centers cover only 36% of the total districts in Jeddah. Thus, increased vaccination sites are needed. It is necessary to establish more vaccination centers to serve the remaining 64% uncovered districts.

### 3.3. Results Validation

**Figure 13:**
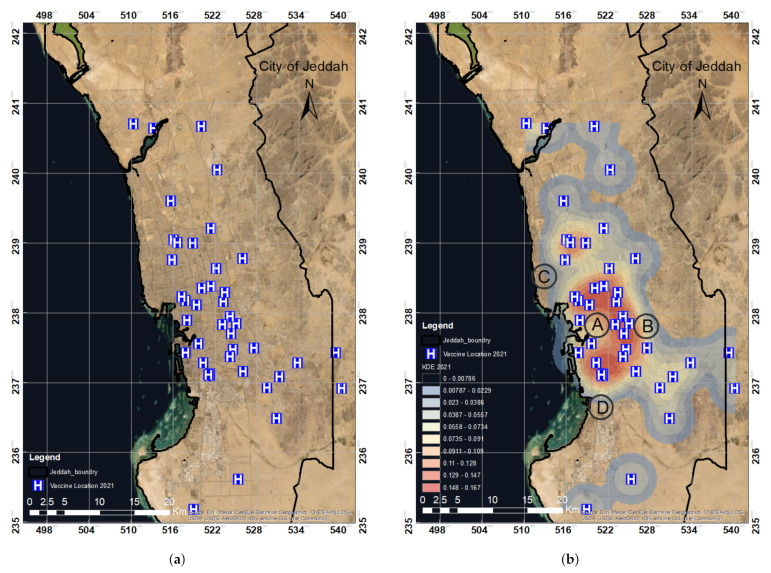
The spatial distribution of the COVID-19 vaccination centers in 2021 in Jeddah (**a**) and the KDE analysis for all 42 vaccination locations in Jeddah in 2021 (**b**), where zone A highlights the city center and zones B and C and D high demand points within uncovered regions. (**a**) Spatial distribution map of the vaccination centers in 2021. (**b**) KDE analysis of vaccination locations in 2021.

In 2021, the Saudi Ministry of Health established 30 new COVID-19 vaccination centers in the city of Jeddah. The locations of these new vaccinati using KDE and Getis–Ord Gi approaches was recommended to enhance the usefulness of the results. However, in this study, we only performed KDE analysis for the new vaccination centers in 2021, mainly because the spatial analysis of the vaccination centers in 2020 showed 95% similarity for both KDE and hotspot analysis. The KDE analysis for the vaccination centers in 2021 indicates that the blue areas are within a radius of 2 km (almost 40 districts), which have full access to vaccines and vaccination centers. The coverage of the vaccination centers was notable and shows enhancements compared to the 2020 coverage of vaccination centers. However, it was observed that a few areas (almost 20 districts) from our study area were still left uncovered by vaccination centers. Figure 13b represents the KDE 2021 analysis for all 42 vaccination locations of Jeddah’s districts.

We note that the vaccination locations are mainly concentrated in the vicinity of the city center, as shown in zone A in Figure 13b. However, the vaccination centers did not cover the east, west, and some of the city’s southern parts, as shown for zones B, C, and D in Figure 13b. The industrial area of Jeddah city is mainly located in the Southern part of the city (zone D). This area is characterized by large populations of industrial workers working in groups; thus, there is a high demand to have vaccination centers. Likewise, zones B and C reflect high demand points within uncovered regions.

**Figure 14:**
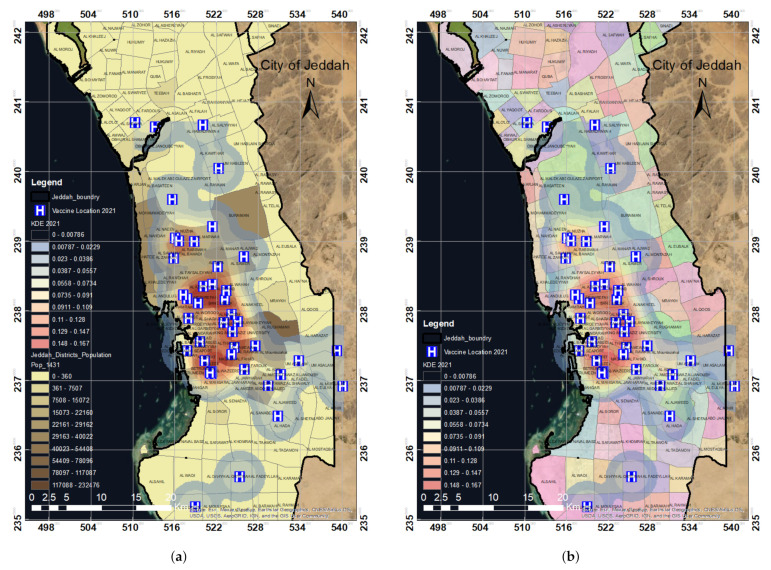
KDE analysis map with respect to (a) population and (b) districts according to the distribution of the COVID-19 vaccination centers in Jeddah in 2021. (**a**) KDE analysis map in relation to population data. (**b**) KDE analysis map in relation to district data.

The KDE analysis of the vaccination centers for the year 2021 was compared with the population, which showed that 42 vaccination centers covered almost all of the highly populated areas. Still, low-to-medium-populated areas remained uncovered. The maximum population coverage is attained by these 42 vaccination centers, as shown in Figure 14b. When comparing the KDE analysis map with the district distribution, we observed that two-thirds of the districts in Jeddah were covered by vaccination centers, as shown in Figure 14a. When we compared the results for both years, we found out that a total of 42 vaccination centers were in operation in 2021, which was a significant improvement on 2020 (only 12). In 2020, more than half of the population was not covered by vaccination centers, but in 2021, two-thirds of the total population of Jeddah was covered. Thus, with the additional 30 vaccination centers in 2021, more people have access to vaccines and vaccination centers.

Based on the spatial analysis of the vaccination centers’ coverage in 2020 and 2021, coverage deficiency would have been addressed earlier if the GIS analysis methods presented in this study had been used by authorities while increasing the number of vaccination centers gradually (for example, in batches of 10 or 15 centers at a time). Of course, the current approaches do not consider other factors in determining the optimal locations of the vaccination centers, such as time constraints and cost. However, the spatial analysis applied in this study would show the missed opportunity between the initial 12 vaccination sites in 2020, the intermediate 22 or 27 sites, and the current 42 vaccination locations in 2021.

## 4. Conclusions

The COVID-19 pandemic negatively impacted many aspects of life, including travel, education, the healthcare system, and the livelihoods of many people. Vaccination is the most effective approach to rapidly eradicating the pandemic’s effects. Due to the rapid spread of the disease, it is necessary to establish vaccination facilities outside hospitals to maximize vaccine coverage. Saudi Arabia, similarly to many countries, developed a COVID-19 vaccination plan to establish vaccination centers across the entire country. In this research work, we examined the significance of the locations of COVID-19 vaccination centers in the city of Jeddah.

KDE analysis was conducted for the vaccination center data for 2020 and 2021 using district and population data for the city of Jeddah. Then, a hotspot analysis was conducted to identify the significant hotspots and coldspots and to determine clustering information and validate KDE analysis. The results revealed a lack of coverage based on the vaccination centers established in 2020. A significant improvement was observed upon repeating the analysis with the additional vaccination centers established in 2021.

This study aimed to inform policymakers and other stakeholders about the regions in Jeddah that are in need of new or additional vaccination facilities. However, other factors, such as time constraints and cost, also need to be considered to optimally determine the locations of vaccination centers. Based on the results of this study, we recommend that the Ministry of Health in Saudi Arabia evaluate the assigned vaccination centers to include less-populated regions to ensure equity and fairness in the vaccine distribution. It is also recommended to add more vaccine centers or reallocate some existing centers in the denser districts to increase coverage in the uncovered sparse regions of Jeddah.

The methods applied in this study could be used in the implementation of a strategic vaccination administration program. The various evaluation maps created using these methods could be used to determine the requirements for establishing vaccination centers. The scientific evaluation of vaccination locations using GIS technologies is crucial, especially during the spread of highly contagious diseases such as COVID-19. The work presented in this study could be a starting point for further research to apply location-allocation algorithms to suggest optimal or nearly optimal sites for vaccine centers in Jeddah to ensure the coverage of the entire population.

## Figures and Tables

**Table 1:** A description of the spatial data for COVID-19 vaccination centers in the city of Jeddah.

Data	GIS Data Type	Data Format	Source
Jeddah base map	Pixel	Raster	Esri, Maxar, GeoEye, Earthstar Geographics,
			CNES/Airbus DS, USDA, USGS, AeroGRID,
			IGN, and the GIS User Community
Vaccination centers	Point	Vector	Saudi Ministry of Health
Population distribution	Polygon	Vector	Jeddah Municipality
Districts distribution	Polygon	Vector	Jeddah Municipality

## Data Availability

Not applicable.
